# Hepatocyte Small Heterodimer Partner Mediates Sex-Specific Effects on Triglyceride Metabolism via Androgen Receptor in Male Mice

**DOI:** 10.3390/metabo11050330

**Published:** 2021-05-20

**Authors:** Brian T. Palmisano, Lin Zhu, Bridget Litts, Andreanna Burman, Sophia Yu, Joshua C. Neuman, Uche Anozie, Thao N. Luu, Emery M. Edington, John M. Stafford

**Affiliations:** 1 Tennessee Valley Health System, Veterans Affairs, Nashville, TN 37212, USA; brian.palmisano@gmail.com (B.T.P.); lin.zhu@vumc.org (L.Z.); sophia.yu@vumc.org (S.Y.); Joshua.c.neuman@gmail.com (J.C.N.); uche.anozie@uvmc.org (U.A.); 2 Department of Molecular Physiology & Biophysics, Vanderbilt University, 2201 W End Ave, Nashville, TN 37235, USA; andreanna.j.burman@Vanderbilt.Edu; 3 Department of Internal Medicine, Stanford Healthcare, Stanford, CA 94304, USA; 4 Department of Medicine, Division of Diabetes, Endocrinology and Metabolism, Vanderbilt University Medical Center, Nashville, TN 37232, USA; bridget.litts@vumc.org (B.L.); thao.ngocluu@gmail.com (T.N.L.); emery.m.edington@vumc.org (E.M.E.)

**Keywords:** sex differences, hypertriglyceridemia, small heterodimer partner, postprandial TG clearance, cardiovascular disease risk

## Abstract

Mechanisms of sex differences in hypertriglyceridemia remain poorly understood. Small heterodimer partner (SHP) is a nuclear receptor that regulates bile acid, glucose, and lipid metabolism. SHP also regulates transcriptional activity of sex hormone receptors and may mediate sex differences in triglyceride (TG) metabolism. Here, we test the hypothesis that hepatic SHP mediates sex differences in TG metabolism using hepatocyte-specific SHP knockout mice. Plasma TGs in wild-type males were higher than in wild-type females and hepatic deletion of SHP lowered plasma TGs in males but not in females, suggesting hepatic SHP mediates plasma TG metabolism in a sex-specific manner. Additionally, hepatic deletion of SHP failed to lower plasma TGs in gonadectomized male mice or in males with knockdown of the liver androgen receptor, suggesting hepatic SHP modifies plasma TG via an androgen receptor pathway. Furthermore, the TG lowering effect of hepatic deletion of SHP was caused by increased clearance of postprandial TG and accompanied with decreased plasma levels of ApoC1, an inhibitor of lipoprotein lipase activity. These data support a role for hepatic SHP in mediating sex-specific effects on plasma TG metabolism through androgen receptor signaling. Understanding how hepatic SHP regulates TG clearance may lead to novel approaches to lower plasma TGs and mitigate cardiovascular disease risk.

## 1. Introduction

Hypertriglyceridemia is associated with adverse cardiovascular outcomes in both men and women. Current therapies aimed at lowering triglycerides (TGs) have proved insufficient in improving cardiovascular disease outcomes [1,2,3,4]. Yet, from human genetic studies, lifelong reductions in plasma TG levels protect against cardiovascular disease [5,6,7,8]. Therefore, understanding pathways regulating plasma TG levels may yield novel targets that protect against cardiovascular disease.

Hypertriglyceridemia can arise from the overproduction of TGs in very-low density lipoprotein (VLDL) particles by the liver or from decreased clearance of circulating TGs. Overproduction of TGs is a hallmark of insulin resistance, which independently increases the risk of cardiovascular disease [9,10]. Some therapies aimed at reducing liver VLDL-TG production have been hampered by the development of fatty liver [11,12]. Impaired TG clearance is associated with cardiovascular disease [13]. Non-fasting plasma TG levels have a stronger association with cardiovascular disease than fasting plasma TG levels, highlighting the importance of postprandial TG clearance in cardiovascular disease risk [14,15,16,17,18]. Therapeutic strategies promoting TG clearance may therefore prevent major adverse cardiovascular events. Several therapies targeting TG clearance are currently in clinical trials [19,20,21].

Understanding differences in lipid metabolism between males and females may reveal mechanisms by which women are protected from cardiovascular disease compared to men. It is well known that premenopausal women are protected from cardiovascular disease compared to age-matched men. Women have more favorable blood lipid profiles including lower triglycerides than men [22,23]. Women have both greater VLDL-TG production and plasma TG clearance than men [23,24,25,26], indicating that enhanced plasma TG clearance likely explains lower TG levels in women compared to men. Mechanisms mediating this sex difference in TG clearance remain incompletely understood.

The nuclear receptor small heterodimer partner (SHP) may mediate sex differences in TG metabolism. SHP represses gene transcription by negative regulation of other nuclear receptors without direct binding to DNA [27,28]. SHP regulates target genes governing bile acid metabolism in the liver [29], as well as genes involved in glucose and lipid metabolism [30,31,32,33]. SHP negatively regulates both Androgen Receptor (AR) and Estrogen Receptor (ER) signaling [34,35], and thus, may mediate sex differences in TG metabolism. In addition to function in the liver, SHP has been shown to function in adipose, testes, and ovaries [32,36,37]. Global knockout models support a role for SHP in glucose and TG metabolism [30,31,36]. However, global SHP knockout alters body weight, adiposity, glucose tolerance, and hepatic steatosis, all of which confound triglyceride metabolism. Furthermore, Hepatic SHP knockout studies have focused predominantly on liver fibrosis [37,38], leaving the role of SHP in plasma TG metabolism unstudied. Since the liver is a major regulator of plasma TG metabolism both directly through TG production and indirectly via the production of secreted proteins that regulate peripheral TG clearance, we investigated the impact of hepatic deletion of SHP on plasma TG metabolism. In this study, we examined plasma TG metabolism in male and female mice with hepatic deletion of SHP (SHP^Δhep^) in comparison to their wild-type littermates. Here, we demonstrate that hepatic knockout of SHP enhances postprandial TG clearance through a pathway specific to males. Our data suggest that hepatic expression of SHP may mediate sex differences in TG metabolism.

## 2. Results

### 2.1. Effect of Hepatic SHP Deletion on Plasma Lipids

**Figure 1:**
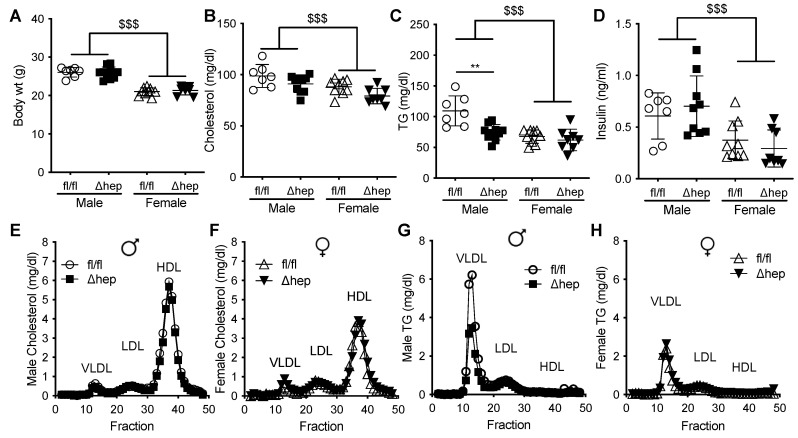
Effect of hepatic deletion of SHP on plasma lipids. (**A**) Body weight of male and female SHP^fl/fl^ and SHP^Δhep^ littermates. (**B**–**D**) Plasma cholesterol (**B**), TG (**C**), and insulin (**D**) of ad lib fed SHP^fl/fl^ and SHP^Δhep^ males and females. (**E**–**H**) FPLC separation of pooled plasma lipoproteins. Cholesterol content of lipoproteins in male (**E**) and female (**F**) SHP^fl/fl^ and SHP^Δhep^ littermates. TG content of lipoproteins in male (**G**) and female (**H**) SHP^fl/fl^ and SHP^Δhep^ littermates. Data shown are mean ± SD, *n* = 7–9/group, ** *p* < 0.01 for genotype difference, ^$$$^ *p* < 0.001 for sex effect (2-way ANOVA with post-hoc comparisons).

To determine whether hepatic SHP impacts sex-specific regulation of TG metabolism, we measured TGs in male and female mice with and without hepatocyte-specific deletion of SHP. There was no difference in body weight between hepatic-SHP knockout mice (SHP^Δhep^) and their wild-type littermates (SHP^fl/fl^) (Figure 1A). Body weight, plasma cholesterol, TGs, and insulin levels were lower in females than in males (Figure 1A–D). Plasma cholesterol was not significantly reduced by hepatic SHP deletion in either sex (Figure 1B,E,F). However, plasma TGs were 30% lower in male SHP^Δhep^ than in male SHP^fl/fl^ mice (78 vs. 109 mg/dL, *p* < 0.01, Figure 1C). The TG reduction by hepatic deletion of SHP in males was primarily within VLDL lipoproteins while the TG content of lipoproteins was similar between SHP^Δhep^ and WT littermates in females (Figure 1G,H). Hepatic deletion of SHP did not alter plasma insulin levels or lipoprotein cholesterols in either sex (Figure 1D–F). These results demonstrate that hepatic deletion of SHP lowered TGs in males to a level similar in females, indicating that hepatic SHP may mediate sex differences in plasma TG metabolism. 

### 2.2. Metabolic Changes after Hepatic Deletion of SHP

**Figure 2:**
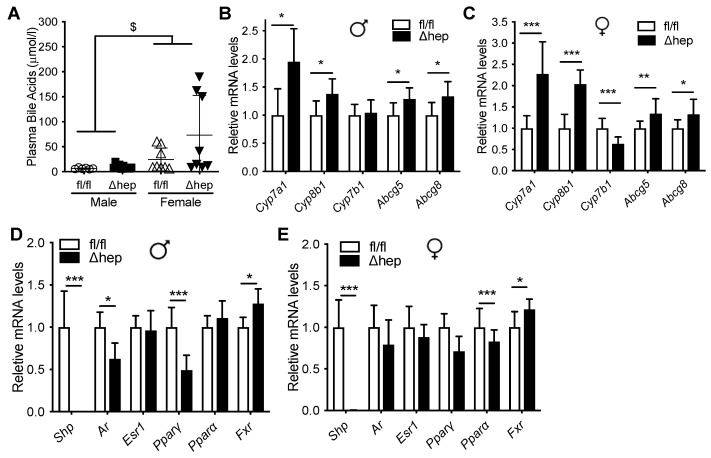
Effect of hepatic deletion of SHP on bile acids. Ad lib chow fed male and female SHP^fl/fl^ and SHP^Δhep^ littermates were used for the study. (**A**) Plasma bile acids. ^$^ *p* < 0.05 for sex effect (2-way ANOVA). (**B**,**C**) Hepatic deletion of SHP increased liver expression bile acid metabolism genes similarly in males (**B**) and females (**C**). (**D**,**E**) Hepatic deletion of SHP reduced expression of Androgen receptor (*Ar*) and *Ppar*γ in males (**D**), but not females (**E**). * *p* < 0.05, ** *p* < 0.01, *** *p* < 0.001 (*t*-test).

Plasma bile acid levels were higher in females and were similar between SHP^Δhep^ and WT controls in both sexes (Figure 2A). mRNA level of *Shp* was reduced more than 1000-fold in SHP^Δhep^ mice in comparison to their WT littermates in both sexes along with the modification of mRNA levels of SHP target genes (Figure 2D,E). As expected from global SHP knockout models [39,40,41,42], hepatic deletion of SHP increased the expression of bile acid metabolic target genes, which was similar in both males and females (Figure 2B,C). 

**Figure 3:**
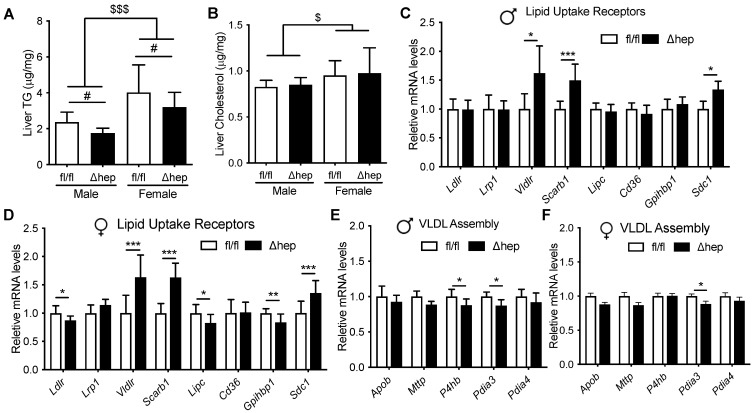
Effect of hepatic deletion of SHP on liver lipid content and metabolism. Ad lib chow fed male and female SHP^fl/fl^ and SHP^Δhep^ littermates were used for the study (*n* = 7–9/group). (**A**,**B**) Liver TG (**A**) and cholesterol (**B**) contents. ^#^ *p* < 0.05 for genotype effect. ^$^ *p* < 0.05, ^$$$^ *p* < 0.001 for sex effect (2-way ANOVA). (**C**,**D**) mRNA levels of liver lipid uptake receptors in SHP^fl/fl^ and SHP^Δhep^ males (**C**) and females (**D**). (**E**,**F**) mRNA expression of VLDL production and assembly genes in males (**E**) and females (**F**). * *p* < 0.05, ** *p* < 0.01, *** *p* < 0.001, (*t*-test).

The liver TG content was higher in females than males and was lower in SHP^Δhep^ than WT controls in both sexes (Figure 3A), which is in agreement with previous reports [31,36,40]. Liver cholesterol content was higher in females than males and was not different between SHP^Δhep^ and SHP^fl/fl^ mice in either sex (Figure 3B). We next investigated the effects of hepatic deletion of SHP on the expression of genes governing liver lipid uptake and genes possibly influencing TG clearance. Hepatic deletion of SHP increased mRNA levels of liver lipid uptake receptors *Vldlr*, *Scarb1*, and *Sdc1* similarly in both males and females (Figure 3C,D). The similar elevation of these genes in both males and females suggests that these genes may not mediate the hypotriglyceridemic effect of hepatic SHP deletion in males. Hepatic deletion of SHP did not significantly change liver mRNA levels of other lipid uptake receptors *Ldlr*, *Lrp1*, *Lipc*, *Cd36*, or *Gpihbp1* in males (Figure 3C). Hepatic deletion of SHP had only modest effects on the expression of genes involved in VLDL assembly in both males and females (Figure 3E,F). Thus, hepatic deletion of SHP altered liver mRNA levels of several genes involved in TG metabolism in males, but these changes were unlikely to explain the sex differences in TG levels. Therefore, liver expression of TG clearance receptors may not explain how hepatic deletion of SHP enhances TG clearance in males. 

### 2.3. Effect of Gonadectomy on Hepatic SHP Regulation of Plasma TGs in Males and Females

**Figure 4:**
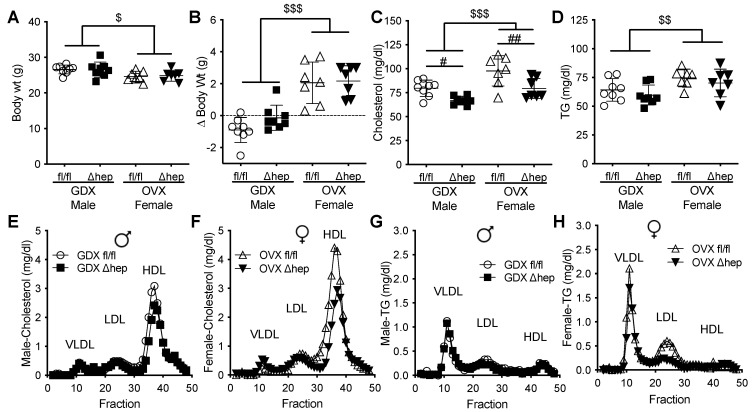
Effect of gonadectomy on hepatic SHP regulation of plasma lipids. Two weeks prior to the study, male and female SHP^fl/fl^ and SHP^Δhep^ littermates underwent surgical gonadectomy (GDX) or ovariectomy (OVX). (**A**,**B**) Body weight (**A**) and body weight change (**B**) after surgery. (**C**,**D**) Plasma cholesterol (**C**) and TG (**D**). (**E**,**F**) Pooled plasma was separated into lipoproteins by FPLC. Cholesterol content of lipoproteins in GDX male (**E**) and OVX female (**F**) mice. (**G**,**H**) TG content of lipoproteins in GDX male (**G**) and OVX female (**H**) mice. Data shown are mean ± SD (*n* = 7–8/group), ^#^ *p* < 0.05 and ^##^ *p* < 0.01 for genotype effects; ^$^ *p* < 0.05, ^$$^ *p* < 0.01, and ^$$$^ *p* < 0.001 for sex effects (2-way ANOVA).

We next hypothesized that male gonadal hormones are involved in hepatic SHP mediated lowering of plasma TGs. To test whether gonadal hormones are required for sex differences in plasma TG metabolism regulated by hepatic SHP, plasma lipids and liver gene expression were measured in surgically gonadectomized males and ovariectomized females with and without hepatic deletion of SHP. Surgical gonadectomy increased the body weight more in female mice than in male mice and minimized the sex differences in body weight and plasma lipids (Figure 4A–D). With surgical gonadectomy, hepatic deletion of SHP lowered plasma cholesterol similarly in both sexes (Figure 4C). In contrast to males with intact gonads, hepatic deletion of SHP failed to lower plasma TGs in males lacking gonadal hormones (Figure 4D vs. Figure 1C). In females lacking gonadal hormones, deletion of hepatic SHP did not alter plasma TGs, which was similar to intact females (Figure 4D vs. Figure 1C). The plasma cholesterol-lowering effect of hepatic SHP deletion in mice lacking gonadal hormones was in HDL fractions in both sexes (Figure 4E,F). In the absence of male sex hormones, hepatic deletion of SHP failed to lower VLDL-TG content as seen in intact males (Figure 4G vs. Figure 1G). In ovariectomized females, hepatic deletion of SHP did not significantly decrease LDL-TG content (Figure 4H). These data suggest that gonadal hormones are required for hepatic deletion of SHP to decrease plasma TGs and VLDL-TG content in males.

### 2.4. Hepatic Deletion of SHP Promotes TG Clearance in Hypertriglyceridemic Mice

**Figure 5:**
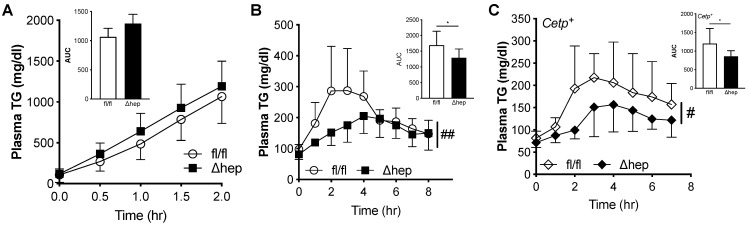
Effect of hepatic deletion of SHP on TG kinetics. (**A**) Plasma TG over time/TG production rate (inset panel) were measured following i.v. injection of Tyloxapol (500 mg/kg) in 3 h fasted male SHP^fl/fl^ and SHP^Δhep^ littermates (2-way Repeated Measures ANOVA, *n* = 7–10/group). (**B**) Mice were fed 20% fructose for 10 weeks. Overnight fasted mice of male SHP^fl/fl^ and SHP^Δhep^ littermates were orally gavaged with olive oil (200 μL/mouse), postprandial TG excursion over 8 h and AUC were measured. (**C**) SHP^Δhep^ mice were crossed with mice expressing the transgene of Cholesteryl Ester Transfer Protein (CETP) to generate CETP^+^/SHP^fl/fl^ and CETP^+^/SHP^Δhep^. Overnight fasted male mice were orally gavaged with olive oil (200 μL/mouse) and postprandial TG excursion were measured. Data shown are mean ± SD (*n* = 6~8/group), # *p* < 0.05 and ## *p* < 0.01 for genotype effect (2-way Repeated Measures ANOVA), * *p* < 0.05 (*t*-test).

Since hepatic deletion of SHP lowered plasma TGs in males, but not females, we measured both TG production and clearance in males to further investigate how hepatic SHP regulates plasma TGs. TG production was measured in fasted mice treated with a lipolytic inhibitor, Tyloxapol (500 mg/kg), and plasma TGs were measured over time. Plasma TG levels over time and TG production rate were similar in SHP^Δhep^ relative to WT littermate controls (Figure 5A). To examine plasma TG clearance, we monitored postprandial TG excursion after an oral fat administration to overnight fasting mice in a fat tolerance assay. We did not see significant increases in plasma TG clearance in SHP^Δhep^ mice compared to SHP^fl/fl^ littermates on chow diet (data not shown), which was likely due to the fast TG clearance rate in mice. We then used mouse models of hypertriglyceridemia to increase the power to detect a difference in TG clearance. First, mice were fed a high fructose diet by supplementing drinking water with 20% fructose for 10 weeks [43]. With fructose supplementation, both SHP^Δhep^ and littermate control mice gained a similar amount of body weight (5.86 ± 3.85 vs. 6.43 ± 2.64 g, *p* = 0.75). Mice with hepatic SHP deletion had lower plasma TG excursion compared to WT littermate controls in the oral fat tolerance test (Figure 5B). Plasma clearance of postprandial TG was increased in SHP^Δhep^ males, as determined by a 24% decrease in area under the curve (*p* < 0.05, Figure 5B). Second, SHP^Δhep^ mice were crossed with mice expressing the Cholesteryl Ester Transfer Protein (CETP) transgene, a genetic model of hypertriglyceridemia [44]. Similar to results in Figure 4B, CETP^+^/SHP^Δhep^ mice had lower postprandial TG excursion relative to CETP^+^/SHP^fl/fl^ controls (Figure 5C). This was consistent with increased plasma TG clearance, as shown by a nearly 30% decrease in area under the curve (*p* < 0.05, Figure 5C). Thus, hepatic deletion of SHP lowers plasma TGs by enhancing plasma TG clearance in males via a male-specific pathway.

### 2.5. Hepatic SHP Regulates Plasma TGs through Androgen Receptor

**Figure 6:**
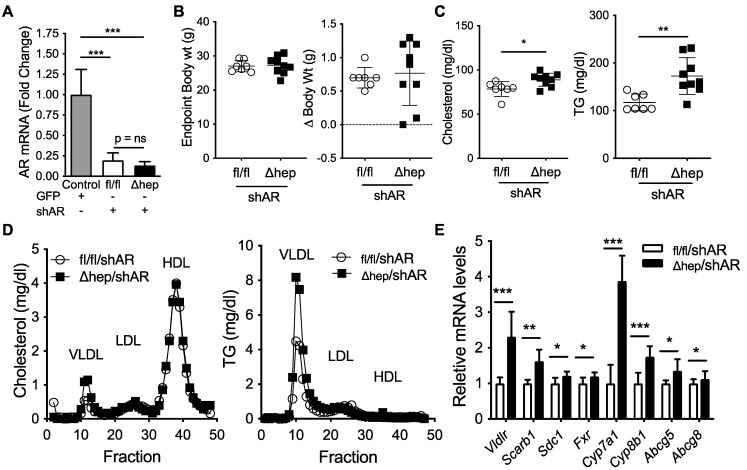
Effect of liver androgen receptor knockdown on hepatic SHP regulation of plasma lipids. Two weeks prior to the study, SHP^fl/fl^ and SHP^Δhep^ littermate male mice were treated with 1.8E12GC of adeno-associated virus serotype 8 (AAV8) containing a short-hairpin RNA to knockdown hepatocyte Androgen Receptor (shAR). (**A**) Liver mRNA levels of *Ar* in SHP^fl/fl^/shAR and SHP^Δhep^/shAR males. Male C57Bl/6 mice treated with AAV8 containing GFP were used as controls. (**B**) Endpoint body weight and body weight change two weeks after viral treatment in SHP^fl/fl^/shAR and SHP^Δhep^/shAR males. (**C**) Plasma cholesterol and TG levels. (**D**) Cholesterol and TG content of FPLC separated lipoproteins of pooled plasma. (**E**) Liver mRNA levels. Data shown are mean ± SD (*n* = 7~9/group), * *p* < 0.05, ** *p* < 0.01, and *** *p* < 0.001 (*t*-test).

To further elucidate the molecular mechanism for the male-specific pathway regulating the sex differences in plasma TG metabolism, we next examined whether liver Androgen Receptor (AR) signaling was required for hepatic SHP to alter plasma TGs in male mice. We hypothesized that hepatic SHP may regulate plasma TGs through liver AR signaling because the TG lowering effect of SHP^Δhep^ was lost in gonadectomized male mice (Figure 4D vs. Figure 2E). To test this hypothesis, we knocked down AR using short hairpin RNA (shAR) delivered via liver trophic adeno-associated viral vector serotype 8 (AAV8, [45]). Two weeks after viral treatment, liver *Ar* mRNA was knocked down >80% (Figure 6A). Body weight and body weight change two weeks after viral injection were similar in SHP^fl/fl^/shAR and SHP^Δhep^/shAR mice (Figure 6B). With *Ar* knockdown, plasma cholesterol was modestly increased in SHP^Δhep^/shAR mice relative to SHP^fl/fl^/shAR mice (Figure 6C). Additionally, with *Ar* knockdown, hepatic deletion of SHP failed to decrease plasma TGs relative to controls; and in fact, plasma TGs were increased with *Ar* knockdown in SHP^Δhep^ mice relative to controls (172 vs. 117 mg/dL, *p* < 0.01, Figure 6C). The changes in cholesterol and TGs were predominantly within VLDL fractions as measured by FPLC separation of lipoproteins (Figure 6D). Thus, liver AR signaling is required for hepatic deletion of SHP to lower plasma TGs and decrease VLDL-TG content in males.

Next, we sought to determine whether AR was required for the TG lowering effect of hepatic deletion of SHP. *Ar* knockdown did not impact body weight (Figure 6A,B); plasma TG and cholesterol levels were higher in SHP^Δhep^/shAR mice than SHP^fl/fl^/shAR mice (Figure 6C). Despite *Ar* knockdown, bile acid metabolic genes were increased in SHP^Δhep^/shAR mice relative to SHP^fl/fl^/shAR mice, which was similar to gonadally intact mice (Figure 6E vs. Figure 2B). Additionally, liver mRNA levels of TG uptake receptors were increased in SHP^Δhep^/shAR mice relative to SHP^fl/fl^/shAR mice despite *Ar* knockdown, which was similar to changes seen in intact males (Figure 6E vs. Figure 3C). Since these mRNA expression changes with hepatic SHP deletion persist despite *Ar* knockdown, these target genes are unlikely to mediate the TG lowering effect of hepatic deletion of SHP in males. 

### 2.6. Hepatic SHP Regulates TG Clearance via ApoC1 and Involves Regulation by Liver AR

**Figure 7:**
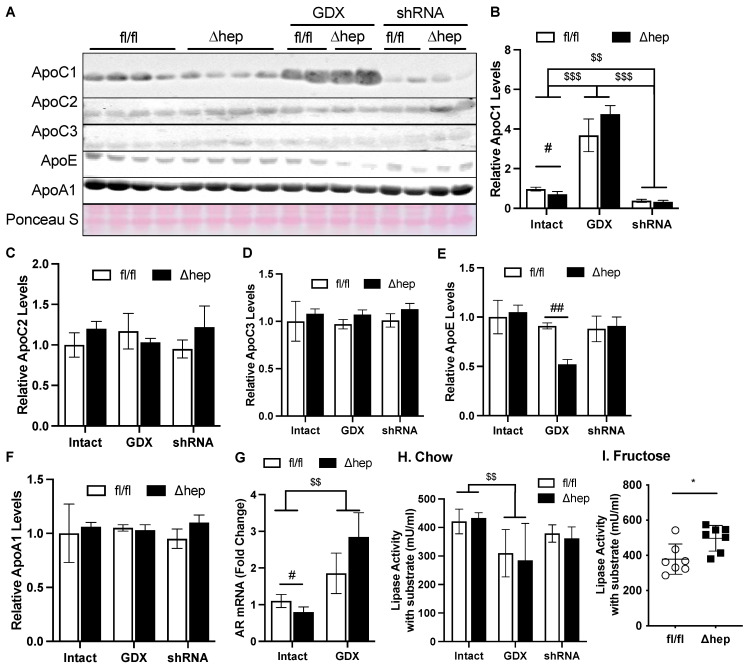
Effect of hepatic deletion of SHP on lipoprotein lipase activities. (**A**–**F**) Plasma ApoC1, ApoC2, ApoC3, ApoE, and ApoA1 levels were determined with immunoblotting and blot quantifications. (**G**) Liver mRNA levels of AR in intact or GDX fl/fl and SHP^Δhep^ mice. (**H**,**I**) Plasma from chow (**H**) and fructose (**I**) fed mice was used to evaluate lipoprotein lipase activity. Data shown are mean ± SD (*n* = 6~8/group), ^#^ *p* < 0.05 and ^##^ *p* < 0.01 for genotype effect, and ^$$^ *p* < 0.01 and ^$$$^ *p* < 0.001 for treatment effects (2-way ANOVA). * *p* < 0.05 (*t*-test).

We hypothesized that the hepatic deletion of SHP may enhance TG clearance through the regulation of genes involved in TG clearance. To further test this hypothesis, we measured plasma levels of apolipoproteins ApoC1, ApoC2, and ApoC3, which regulate peripheral lipase activity. Hepatic deletion of SHP decreased plasma ApoC1, but this effect was lost with gonadectomy (Figure 7A,B). Additionally, in the presence of gonadal hormones while liver *Ar* was knocked down, hepatic SHP deletion failed to lower plasma ApoC1 (Figure 7A,B). Hepatic deletion of SHP did not affect plasma levels of ApoC2 or ApoC3, other regulators for lipase activity (Figure 7A–D). Plasma ApoE was only decreased by hepatic deletion of SHP in the absence of gonadal hormones, while plasma ApoA1 was not altered between experimental groups. To further understand why plasma ApoC1 was increased in gonadectomized mice and decreased in shAR mice, we measured mRNA levels of liver *Ar*. Liver *Ar* mRNA was decreased by hepatic deletion of SHP and increased by the removal of gonadal hormone in male mice, falling in the same pattern of changes for plasma ApoC1 (Figure 7A,B,G).

We next examined lipase activities and if they were impacted by the ApoC1 changes induced by hepatic SHP deletion. Plasma ApoC1 inhibits lipase activity and TG clearance [46,47]. The plasma from SHP^Δhep^ mice did not significantly increase lipase activity relative to the plasma from SHP^fl/fl^ littermate controls (Figure 7H), which is in line with our observation that TG clearance was not different in mice fed a chow diet (data now shown). When hypertriglyceridemia was induced by a fructose administration, the decrease in plasma ApoC1 caused by hepatic deletion of SHP was associated with increased lipase activity and faster TG clearance in SHP^Δhep^ mice than in SHP^fl/fl^ mice (Figure 5B and Figure 7I). Gonadectomy was associated with a four-to-six-fold increase in plasma ApoC1 levels, which was associated with lower plasma lipase activity relative to intact mice (Figure 7H). After gonadectomy, hepatic deletion of SHP did not lower ApoC1 or impact lipase activity (Figure 7A,B,H). Our results together indicate that hepatic deletion of SHP may enhance plasma TG clearance in males by suppressing ApoC1 and subsequently enhancing lipase activity.

## 3. Discussion

**Figure 8:**
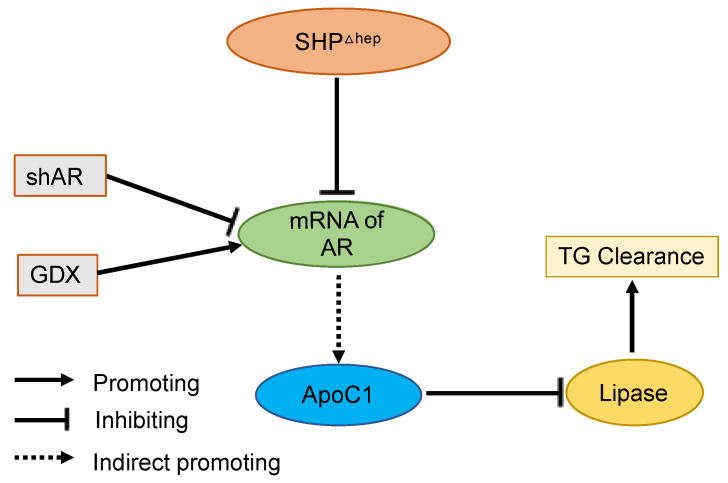
Graphic Model. Hepatic deletion of SHP (SHP^Δhep^) leads to the downregulation of liver androgen receptor (AR) mRNA, decreased plasma ApoC1 protein levels, increased lipase activity, and increased triglyceride (TG) clearance. Reduction of liver AR mRNA by shRNA or androgen levels by gonadectomy (GDX) respectively decreases or increases ApoC1 protein levels and blunts the effect of hepatic deletion of SHP on blood TG levels.

Here, we demonstrate that liver SHP regulates plasma TGs via a male-specific pathway. Plasma TGs were higher in male mice than female mice, and hepatic deletion of SHP lowered plasma TGs and VLDL-TG content in males but not in females. Removal of male sex hormones abolished plasma TGs lowering effect of hepatic SHP deletion in males. Hepatic deletion of SHP altered the expression of several targets of liver TG metabolism, yet the changes were similar in males and females, arguing against their mechanistic importance since the TG lowering was only seen in males. Hepatic deletion of SHP reduced plasma TGs by decreasing the lipase inhibitor ApoC1 in plasma and increasing postprandial TG clearance, which was associated with decreased *Ar* mRNA levels in the liver. Furthermore, Hepatic deletion of SHP failed to further decrease ApoC1 and lower plasma TGs when liver AR was knocked down. Taken together, these data indicate that hepatic SHP regulates plasma TGs by modifying plasma ApoC1 activity and postprandial TG clearance in male mice through a pathway associated with liver AR mRNA expression (Figure 8). 

Our results suggest that a male-specific pathway contributes to sex differences in plasma TG metabolism. The elevated plasma TGs may contribute to an increased risk of cardiovascular disease in men vs. women [23,24,25,26]; however, the mechanisms for the increased risk in males are not fully understood. While estrogen regulation of lipid metabolism has been well studied [48,49], male sex hormone-specific regulation of lipid metabolism needs to be better explored. Testosterone has been an attractive explanation for the increased cardiovascular risk and plasma TGs in men, although testosterone supplementation in healthy men or testosterone replacement in men with hypogonadal hypogonadism has shown minimal effects on plasma TG levels [50,51,52]. In the current study using male and female mice, we show that hepatic SHP may mediate sex differences in plasma TGs in males but not in females. Prior studies may have missed the sex specificity of the hepatic SHP pathway in plasma lipid metabolism since only male animals were used [30,33,46,53] or there was little information available for females [31]. Although SHP may regulate the expression of estrogen receptor and consequently modify plasma and liver TGs [52,54,55], we did not find a significant impact of hepatic SHP on TG metabolism in females that were fed a chow diet. Multiple female-specific pathways may exist to promote TG delivery into peripheral tissues due to the evolutionary pressure, which results in low plasma TG levels in females [23,24,25,26]. Although it has been reported that estrogens may modify SHP expression [54], we did not observe significant differences in hepatic SHP mRNA levels between wild type male and female mice (data not shown), indicating that the lower TG levels in females were independent of hepatic SHP. Additionally, ovariectomy in female mice did not unmask the effect of hepatic SHP on plasma TGs. Thus, hepatic SHP regulates plasma TGs in a male-specific pathway. 

Data presented here support a male-specific pathway that hepatic SHP regulates postprandial plasma TG clearance. Prior global SHP knockout models have shown an increase [31,56], a decrease [30,40], or no effect on plasma TGs [32,33,46,57], indicating that the model used for the study may be of particular importance. Additionally, SHP has been shown to have adipose-specific functions [32,36,37], suggesting that tissue-specific functions of SHP have disparate effects on TG metabolism. Using hepatic specific knockout of SHP, our data implicates that hepatic SHP increases plasma TGs in males by impairing TG clearance without changing TG production. It does not seem that lipid catabolism contributes to the decreases in plasma TGs because the energy expenditure and RQ were similar between hepatic SHP knockout male mice and their wild-type male littermates when they were fed a high-fat diet in a separate study (unpublished data). We used chow-fed mice in the current study to avoid the confounding effects of insulin resistance and obesity on plasma TG clearance. We observed differences in plasma TGs in the ad lib fed, indicating that TG clearance during the postprandial state is regulated by hepatic SHP. This was also supported by the results that the decreased plasma TGs were contributed to decreases in VLDL-TG content in hepatic SHP knockout mice. TG clearance during fasting evaluated by fat tolerance test was not significantly different in chow-fed mice (data not shown). However, hepatic SHP deletion significantly promoted TG clearance during fasting when hypertriglyceridemia was induced either by fructose diet or in a genetic hypertriglyceridemic background (Figure 5). Our data highlight the role of hepatic SHP in plasma TG metabolism and TG clearance. 

This work demonstrates a novel pathway contributing to elevated TGs in males, which is governed by hepatic SHP and requires liver AR signaling. We show that plasma TGs decreased by hepatic deletion of SHP was associated with a downregulation of liver *Ar* mRNA in male mice. The hypotriglyceridemic effect of hepatic SHP deletion was lost in GDX and shAR liver *Ar* knockdown mice, suggesting the male sex hormones and liver AR are involved in hepatic SHP regulation of plasma TG. Several liver apolipoproteins are known to regulate plasma TG clearance (reviewed [58]). Of these proteins, we show that ApoC1 may play a role in plasma TG clearance mediated by hepatic SHP in males. We observed that changes of plasma ApoC1 and lipase activities fell in the same pattern of changes of liver *Ar* mRNA levels in the current study. ApoC1 is a 57 AA protein and acts on lipoprotein receptors by inhibiting ApoE’s binding to suppress tissue lipid uptake [46,59]. With regard to lipid lipase activities for plasma TG clearance, ApoC1 inhibits the activities of multiple lipases including lipoprotein lipase, hepatic lipase, and phospholipase A2 [53,55,60]. Transgenic mice of ApoC1 have impaired plasma TG clearance and severe hypertriglyceridemia [57]. ApoC1 is highly expressed in the liver and a lesser degree in the adrenal glands [46]. We show that plasma ApoC1 levels are decreased by hepatic deletion of SHP, which was associated with a downregulation of liver AR mRNA. Using the mouse model with the liver *Ar* knockdown, we show that AR was required for liver SHP to regulate plasma ApoC1 levels. In the absence of male gonadal hormones by gonadectomy, hepatic SHP failed to regulate ApoC1 levels. Interestingly, gonadectomy increased ApoC1 levels, associated with compensatory upregulation of liver *Ar* mRNA. Data presented here highlight a novel pathway that may contribute to sex differences in TG metabolism and, potentially, risk of cardiovascular disease. 

Our study implicates hepatic SHP in sex differences in TG metabolism. Information on the regulatory mechanism for ApoC1 by androgen receptor is scarce in the literature but of great interest in future studies. Further understanding of this pathway may further elucidate mechanisms of sex differences in lipid metabolism and potentially cardiovascular disease risk. A better understanding of sex differences in lipid metabolism and cardiovascular disease risk may lead to additional therapies that mitigate cardiovascular disease in both men and women.

## 4. Materials and Methods

### 4.1. Animals, Genotyping, and Viral Knockdown

All mouse experiments were approved by the Vanderbilt University Institutional Animal Care and Use Committee (The animal welfare assurance number is A-3227-01). Mice were housed in 12-h light/dark cycles in temperature and humidity-controlled facilities with ad-libitum access to chow diet and water. Mice with homozygous floxed *Shp* allele were crossed with mice with Cre recombinase under control of the albumin promoter to generate hepatocyte-specific knockout of *Shp* (Δhep; SHP^Δhep^). These mice were generously provided by Dr. David Moore at Baylor, were back-crossed on a C57BL/6J background, and have been previously described [44]. Floxed littermates lacking Cre were controls (fl/fl, SHP^fl/fl^) in all experiments. All strains were backcrossed at least 10 generations onto the C57BL/6J background (Jax Stock No: 000664). Genotyping was done from tail DNA. Detection of the SHP^flox^ allele was done using a multiplex reaction with primers SHP-F (GCCTTTAACTCAAGTACTAGGGAGGCAG), SHP-R1 (CTACCCAGAGCGACATGGTGAGAC), and SHP-R2 (AGTTGTGTCTGGTTCCTGACCTTGG). Once the SHP^flox^ allele was bred to homozygosity, only detection of Cre recombinase was done. Cre recombinase was detected using primers Cre-F (GAACCTGATGGACATGTTCAGG) and Cre-R (AGTGCGTTCGAACGCTAGAGCCTGT) in a multiplex reaction with control primers Myogenin-F (TTACGTCCATCGTGGACAGC) and Myogenin-R (TGGGCTGGGTGTTAGCCTTA). Mice were 14 ± 2 weeks old to ensure complete sexual development. All animals were sacrificed between 8 and 11 a.m. to minimize circadian variation in gene expression. Androgen receptor knockdown was done using an adeno-associated virus, serotype 8 (AAV8) containing short-hairpin RNA (shRNA) specific to mouse androgen receptor under control of a U6 promoter with a GFP reporter under control of a CMV promoter (shADV-252885, Vector Biolabs, Philadelphia, PA, USA). AAV8 was chosen for its liver tropism [45]. 1.8E12 GC/mouse was injected intravenously under brief isoflurane anesthesia. We have previously shown that this dose of viral vector did not affect plasma TG metabolism [61].

### 4.2. Gonadectomy and Ovariectomy Surgery

In males, midline scrotal excision was used to externalize testes, which were ligated with single interrupted 5-0 suture and excised. Skin was closed with 9 mm autoclips. In females, midline dorsal skin incision was made followed by two lateral incisions of the dorsal peritoneal wall to remove each ovary. Single simple interrupted stitches with 5-0 suture were used to close peritoneal incisions and 9 mm autoclips were used to close the dorsal skin incision. In all mice, prophylactic antibiotic was given once postoperatively (ceftriaxone, 15–25 mg/kg) and analgesic was given once preoperatively and every 24 h postoperatively for 2 days (ketoprofen, 5–10 mg/kg). Mice were housed individually for 3–4 days to allow sufficient wound healing and then returned to original littermate housing. Mice were allowed to recover for 2 weeks prior to the study. Mice weighing less than 90% of their pre-surgical mass were euthanized and excluded. 

### 4.3. Lipid and Lipoprotein Analysis

Plasma TG and cholesterol were measured using colorimetric kits (Infinity, ThermoFisher, Asheville, NC, USA). Lipoproteins were separated by fast-performance liquid chromatography (FPLC, Superose6 column, GE Healthcare, Fort Myers, FL, USA) from pooled plasma samples (150 μL total pooled plasma used). Liver TG content and liver cholesterol content were determined by the Vanderbilt Hormone Assay Core. Briefly, 50–100 mg liver was Folch extracted and separated by thin layer chromatography, which was then analyzed by gas chromatography with internal standards used to control for extraction efficiency.

### 4.4. In Vivo TG Production and Clearance 

TG production was measured in 3-h fasted mice after intravenous administration of Triton WR-1339 (500 mg/kg, Sigma, Saint Louis, MO, USA), an inhibitor of lipolysis. Postprandial TG clearance was measured after 10 weeks of ad libitum access to 20% fructose supplementation to drinking water or with transgenic expression of Cholesteryl Ester Transfer Protein (CETP), a genetic model of impaired TG clearance [44]. Transgenic CETP mice (C57BL/6-Tg(CETP)UCTP20Pnu/J, Strain: 001929, Jackson Laboratories), were bred with Δhep mice to generate fl/fl (CETP^+^/SHP^fl/fl^) and Δhep (CETP^+^/SHP^Δhep^) mice. TG clearance was measured in 12-h fasted mice after oral gavage of olive oil (200 μL/mouse) with serial tail blood samples over 7–8 h. 

### 4.5. Western Blotting

Four microliters of serum/plasma were used for immunoblotting to quantify plasma levels of ApoC1, ApoC2, and ApoC3 with a protocol we published previously [62]. Ponceau S staining was used for normalization. Rabbit anti-mouse ApoC1 antibody was from Abcam (ab231570); rabbit anti-mouse ApoC2 (PA5102480) and anti-mouse ApoC3 (PA578802) antibodies were from Invitrogen (ThermoFisher, Asheville, NC, USA), rabbit anti-mouse ApoA1 (K23100R) and ApoE (K23500R) antibodies were from Meridian (Memphis, TN, USA). IRDye-800CW goat anti-rabbit IgG was from LI-COR Biosciences (ThermoFisher, Asheville, NC, USA).

### 4.6. Blood Lipase Activities 

To quantify the regulatory activity of plasma lipase regulators including ApoC1, ApoC2, and ApoC3, we used a Lipase Assay Kit (Cat#STA-610, Cell Biolabs, San Diego, CA, USA) with a 2-step protocol. Briefly, in step A, two microliters of each plasma sample were used to measure blood total lipase activity following the manufacture’s protocol (data not shown). In step B, two microliters of each plasma sample were used to measure lipase activity in the presence of 1.4 mUnit of LPL from Cell Biolabs. Data from step B are shown for regulatory activity of plasma lipase regulators.

### 4.7. Liver and Adipose mRNA Quantification 

Liver and adipose samples were stored in RNA-Later at 4 °C overnight and then −20 °C according to the manufacturer’s instructions (ThermoFisher, Asheville, NC, USA). A small piece of tissue was bead homogenized and mRNA was isolated according to the manufacturer’s instructions (Trizol, Qiagen, Germantown, MD, USA). Complementary DNA was synthesized from 1 μg of mRNA (iScript, Bio-Rad, Hercules, CA, USA). RT-PCR was done in triplicate from 10 ng cDNA (JumpStart Taq ReadyMix, Sigma, Saint Louis, MO, USA). All primers Table 1) were validated using a melting curve and annealing temperatures were optimized using gradient RT-PCR. Expression of all genes was calculated using the efficiency corrected Pfaffl method with normalization to cyclophilin A (*Ppia*) [63]. Efficiency was measured from amplification curves by the program LinRegPCR according to Ruijter et al [64].

### 4.8. Statistical Analysis 

Data are summarized using mean and standard deviation. Statistical tests between two groups were analyzed by unpaired Student’s *t*-test. Data with more than two groups were analyzed by 1-way ANOVA with Bonferroni post-hoc comparisons. Repeated measures 1-way ANOVA was used for measures of plasma TG over time with Bonferroni post-test comparisons. Genotype or sex effects were determined by 2-way ANOVA. *p*-values of <0.05 were considered statistically significant.

## Figures and Tables

**Table 1:** Primer probes for qPCR.

Gene	Forward Primer	Reverse Primer
*Abcg5*	CCTGCAGAGCGACGTTTTTC	GCATCGCTGTGTATCGCAAC
*Abcg8*	TGGATAGTGCCTGCATGGATC	AATTGAATCTGCATCAGCCCC
*Apob*	GCCCATTGTGGACAAGTTGATC	CCAGGACTTGGAGGTCTTGGA
*Ar*	GGACCATGTTTTACCCATCG	TCGTTTCTGCTGGCACATAG
*Cd36*	TGGCCTTACTTGGGATTGG	CCAGTGTATATGTAGGCTCATCCA
*Cyp7a1*	AGCAACTAAACAACCTGCCAGTACTA	GTCCGGATATTCAAGGATGCA
*Cyp7b1*	TAGCCCTCTTTCCTCCACTCATA	GAACCGATCGAACCTAAATTCCT
*Cyp8b1*	GCCTTCAAGTATGATCGGTTCCT	GATCTTCTTGCCCGACTTGTAGA
*Fxr*	TCCGGACATTCAACCATCAC	TCACTGCACATCCCAGATCTC
*Gpihbp1*	TACCTACTCCATGTGGTGTACTG	AGGATGTCTAGTCCCACTTTCC
*Ldlr*	GCATCAGCTTGGACAAGGTGT	GGGAACAGCCACCATTGTTG
*Lipc*	GACGGGAAGAACAAGATTGGAA	GCATCATCAGGAGAAAGG
*Lrp1*	TCAGACGAGCCTCCAGACTGT	ACAGATGAAGGCAGGGTTGGT
*Mttp*	CAAGCTCACGTACTCCACTGAAG	TCATCATCACCATCAGGATTCCT
*P4hb*	GCCGCAAAACTGAAGGCAG	GGTAGCCACGGACACCATAC
*Pdia3*	CGCCTCCGATGTGTTGGAA	CAGTGCAATCCACCTTTGCTAA
*Pdia4*	TCCCATTGCTGTAGCGAAGAT	GGGGTAGCCACTCACATCAAAT
*Pparα*	TATTCGGCTGAAGCTGGTGTAC	CTGGCATTTGTTCCGGTTCT
*Ppia*	CGATGACGAGCCCTTGG	TCTGCTGTCTTTGGAACTTTGTC
*Scarb1*	TCAGAAGCTGTTCTTGGTCTGAAC	GTTCATGGGGATCCCAGTGA
*Sdc1*	CTTTGTCACGGCAGACACCTT	GACAGAGGTAAAAGCAGTCTCG
*Shp*	CGATCCTCTTCAACCCAGATG	AGGGCTCCAAGACTTCACACA
*Vldlr*	CCACAGCAGTATCAGAAGTCAGTGT	CACCTACTGCTGCCATCACTAAGA

## Data Availability

The data presented in this study are available in article.
